# Dog Training Intervention Shows Social-Cognitive Change in the Journals of Incarcerated Youth

**DOI:** 10.3389/fvets.2018.00302

**Published:** 2018-12-11

**Authors:** Tiffany Syzmanski, Rita J. Casey, Amy Johnson, Annmarie Cano, Dana Albright, Nicholas P. Seivert

**Affiliations:** ^1^Psychology, Wayne State University, Detroit, MI, United States; ^2^School of Nursing, Oakland University, Rochester, MI, United States

**Keywords:** dogs, dog training, incarcerated youth, journaling, animal-assisted treatment

## Abstract

There is limited research assessing the effectiveness of Animal-Assisted Therapy in at-risk adolescent populations. In a recent study, 138 adjudicated adolescents participated in a randomized controlled trial of an animal-assisted intervention, in which participants either trained shelter dogs (Teacher's Pet group) or walked the dogs (control group), with both groups participating in classroom work related to dogs (1). Journal writing was a part of class activities for all youth in the study. Conventional assessments of youth behavior made by staff or youth themselves did not demonstrate the expected differences between the groups favoring the dog training group, as youth in both groups showed a significant increase in staff and youth rated internalizing behavior problems and empathy from the beginning to the end of the project (1). However, subsequent analysis of the journal content from 73 of the adjudicated youth reported here, did reveal significant differences between treatment and control groups, favoring the Teacher's Pet group. Youth participating in the dog training intervention showed through their journal writing greater social-cognitive growth, more attachment, and more positive attitudes toward the animal-assisted intervention compared to youth in the control group. The 73 youth whose journals were available were very similar to youth in the larger group. Their results illustrate that journaling can be a useful method of assessing effects of similar animal-assisted interventions for at-risk youth. Writing done by youth receiving therapy appeared to promote self-reflection, desirable cognitive change, and prosocial attitudes that may signify improving quality of life for such youth. The expressive writing of participants could reveal important effects of treatment beyond the behavioral changes that are often the targeted outcomes of animal-assisted interventions.

## Introduction

This study analyzed the content of journals kept by incarcerated youth who participated in a randomized controlled trial of an animal-assisted therapy, known as Teacher's Pet (1). Incarcerated youth in the United States are highly likely to have a psychiatric disorder (2), a predictor of recidivism (3). Effective treatment of at-risk youth is crucial not only to reduce the risk of recidivism in adulthood, but also to improve their quality of life (4–6). This project determined whether journal entries made during an animal-assisted treatment of detained youth could be analyzed meaningfully, and if so, whether their content provided insight into potential positive effects of the treatment.

Youth are placed in detention for many reasons. About a quarter of youth engage in severe crimes, such as homicide, robbery, or aggravated and sexual assault (7). However, the vast majority of imprisoned youth engage in less serious crimes such as theft, burglary, substance abuse, simple assault, weapon possession, running away, particularly from foster homes (8), or truancy, etc.

The majority of incarcerated youth in the United States have been given diagnoses or are likely to meet diagnostic criteria for some form of mental illness. Teplin et al. (4) found that the most common disorders diagnosed were (in terms of male and female prevalence, respectively), substance use disorders (50.7 and 46.8%) conduct and oppositional defiant disorder (41.4 and 45.6%), and high rates of other disorders characterized by internalizing symptoms such as anxiety (21.3 and 30.8%) and other mood problems (18.7 and 27.6%) (4). Other studies with detained youth have found similar rates of mental disorders (5, 6).

Given the high prevalence of psychopathology in this population, it is important that treatments are available for these youth, to address such problems. Treatment regimens for incarcerated youth most commonly focus on substance abuse. However, interventions with those targets are not sufficient to meet the needs of youth with other mental disorders and multiple diagnoses (4, 6, 9, 10). Nevertheless, very few facilities implement appropriate treatments for incarcerated youth. Wilson and Lipsey (11) examined the effects of different treatments of adjudicated youth aimed at reducing recidivism, finding that overall, they reduced recidivism rates by 12%. The most effective programs focused on building social and communication skills via reinforcement in learning adaptive interactions (12). Mixed results were found from other treatment methods, such as cognitive behavioral approaches and vocational training.

More recently, interaction with animals has become a way to provide treatment to youth with serious psychological difficulties, including incarcerated youth. Such treatments hold promise of being possibly easy to implement, and attractive as novel, highly liked intervention that many youth will enjoy (13). Such interventions have ranged widely in terms of the animals used as the central aspect of the treatment, as well as the kind of target problems and participants who are included (14).

Inexpensive methods for examining effects of animal assisted treatment, such as expressive writing, could be integrated into treatment programming, in order to show outcomes of animal-assisted interventions for incarcerated, high-risk youth. Journaling is a form of expressive writing that is typically done at regular, frequent intervals. Individuals doing journaling record their thoughts, feelings, and experiences about their life. It is an activity that has shown therapeutic as well as learning benefits (15–18), and can be used as either a treatment in itself, or as an adjunct or way to examine the outcomes of treatments. It is a form of self-reflection that can facilitate self-awareness, personal growth, insight into emotions and behaviors, and aid in restructuring persons' thoughts about their experiences.

Although expressive writing is considered to be helpful in adult populations, very few studies have focused on the benefits of expressive writing for youth. Journaling could potentially be a very useful activity for adolescents in that it is an engaging and creative, especially in the age of social media in which youth are accustomed to sharing their thoughts and actions with their peers via the internet, often through writing (19, 20). Expressive writing could be a useful method for examining effects of treatment given to incarcerated youth, in that writing is inexpensive to implement, and can also help fill the passage of time for youth whose incarceration makes many ordinary youth activities beyond the range of possibility. Writing down thoughts and emotions about life events, particularly those that are stressful, can aid youth in expressing and understanding their deepest emotions and forming a deeper understanding of their life experiences. Writing can ultimately help to reduce stress by providing a form of written disclosure (17, 21). Keeping a journal can also help youth develop a sense of meaning in life, show their understanding of life events, and provide a window into critical thinking skills, changed self-perceptions, and better skills for coping with difficult emotions (15, 22).

Although more research is needed on expressive writing with adolescents, it is a promising activity for teens, particularly if it can help provide insight into those who are considered at-risk. Adolescents, particularly ones with behavioral problems, can use journaling to record their reactions to treatment and reflect on them (23). Journaling can also potentially aid in revealing youths' self-awareness of life experiences, which can, in turn, increase empathy toward others and ultimately, improve interpersonal relationships (23, 24). Thus, journaling among adolescents can facilitate examination of personal growth, self-awareness, and insight, especially since adolescence is a big transitional period in emotional development from childhood to adult life (20).

Expressive writing, with its emphasis on communication of emotions, thus presents a potentially welcome and cost-effective approach for assessing treatment outcomes for incarcerated youth. Through writing about their own emotions and thoughts associated with life events, they have an opportunity to develop their perspective taking, as well as improving insight into their own behavior and generating a sense of self-efficacy in their actions (25). Furthermore, when expressive writing through journaling is done as a way to look at the effects of a time-bound intervention, such as the animal-assisted treatment reported in this project, such writing has the potential to be a valuable asset to that intervention, allowing a look at changes that may be difficult to capture through additional outcome measures focused primarily on behavior ratings. A recent meta-analysis of effects of treatments given to youth with conduct disorder (26) found that youth self-ratings were typically non-significant, and similar ratings from parents and teachers showed small to moderate effects, mostly from parents. Thus journaling might offer a way to examine treatment outcomes of youth that will not be observed in self-ratings of youth behavior.

The original project from which the current study was drawn (1), was an animal-assisted therapy intervention with incarcerated youth, in which interaction with shelter dogs was a crucial part of the intervention. Participants in a dog-training intervention (known as Teacher's Pet) designed to teach youth how to train undersocialized dogs, were expected to improve in human social, emotional, and behavioral functioning. A control group, whose principal activity consisted of walking dogs for the same amount of time with dogs as the treatment group, were expected to have less positive outcomes than the dog-training youth,. However, behavior ratings from staff members at the facilities as well as self-ratings of the youth did not show positive changes as a function of group assignment. Rather, youth in both the Teacher's Pet intervention and the dog walking control group demonstrated increases in internalizing behaviors. In addition, all youth increased in empathy, with no differences by group assignment.

This project analyzed journal content of incarcerated teens who participated in that animal-assisted therapy (AAT) (1). We wanted to see whether journal writing would show outcomes that demonstrated differences between treatment and control conditions, consisting of youths' thoughts concerning social relationships and attitudes during the AAT. Thus, this project analyzed the journals produced by incarcerated youth who were participating in an animal-assisted therapy compared to journals of youth in a control condition. We looked for meaningful patterns of content in the youths' writing that would vary according to participants' group assignment to either the experimental treatment or the control condition.

Specifically, we hypothesized that the pattern of writing content would covary with participant group membership in ways showed more positive outcomes for the experimental condition. If this expectation was correct, journal writing of the experimental group's participants would show more positive signs of social cognition and attitudes than were observed in the journal writing of youth in the control group.

## Materials and Methods

### Participants

Participants in this project were drawn from the larger Teacher's Pet Research Study (1). That project consisted of 138 adolescents in two county juvenile detention centers in Southeast Michigan. By the end of the last cohort in June 2014, 73 participant journals were collected and identified as to particular youth. The missing journals were primarily unavailable because initially, the journal writing was not thought of as reflecting any meaningful outcome of the intervention, thus no plans were initially made to collect youth journals. However, a quick review of journals from some of the earliest participants in the project showed some interesting content, so journals were given code numbers to associate them with other data collected from individual participants. This took some time to implement, thus some youth took their writing materials with them when they left the facility they were in, before their journals could be copied. Other journals were copied, but without the needed identification to associate them with other individual data.

The youth whose journals were available to this project, were very similar in overall characteristics to the larger group from which they were drawn. Participants were mostly male (72.5%); 58.9% of participants were in the Teacher's Pet group (*N* = 43), and 41.1% of participants were in the Dog walking control group (*N* = 30) (see Table 1 for overall characteristics of the youth whose journals were available for this study). The background of the youth with journals available for this study were very similar to that of the larger group. About one-quarter of the participants had a history of parental abuse or neglect, or a history in the foster care system. Two-thirds had a psychiatric diagnosis and/or had been in treatment for one. Ethnicity and gender were not specifically controlled for this study; rather, these characteristics reflected the general makeup of adolescents in the juvenile detention centers that were involved in the program. The percentages by ethnicity were very similar in the 73 youth in this report compared to the larger group. Analyses of the distribution of these characteristics in the larger and smaller groups showed no significant differences in the distribution of ethnicity (*X*^2^ = 0.385, *p* = 0.55) or gender (*X*^2^ = 0.212, *p* = 0.66) between the larger set of 138 youth and the 73 youth whose journals were analyzed for this study.

**Table 1:** Characteristics of participants.

**Variable**	**% of participants**	***N***	**TP**	**DW**
**ETHNICITY**				
White/Caucasian	49.3	36		
Black/African American	39.7	29		
Hispanic/Latino	5.5	4		
Other	5.5	4		
**SEX**				
Male	72.6	53	26	27
Female	27.4	20	17	3
Site 1	49.3	36	26	10
Site 2	50.7	37	17	20
**TOTALS**				
Treatment (TP)	58.9	43		
Control (DW)	41.1	30		
Overall	100.0	73		

*TP, Teacher's Pet (Intervention); DW, Dog walking (Control)*.

### Procedure

Participants in each cohort were randomly assigned to either an animal assisted therapy group (the experimental group, known as Teacher's Pet) or a dog walking control group. The Teacher's Pet Program is an animal assisted therapy program (AAT) that had already been carried out at both facilities prior to becoming the center of this research project. Those activities were familiar to center staff but had not been executed in comparison to any other intervention or comparison activity in the past. In the experimental group's activity, youth were instructed to train undersocialized dogs in order to make the dogs more suitable for adoption out of the shelters from which they came. Participants in the control group walked assigned dogs but did not teach them. Both youth who were dog walkers and youth who participated in Teachers' Pet training worked with the same dogs. Somewhat more youth were assigned to the Experimental (Teacher's Pet) group than the Control, dog walking group, because of facility staff requests, scheduling, and the desire to populate the Experimental group because of the small sample. This limitation is discussed further in the Discussion section. We note, however, that the sizes of treatment and control groups were more similar across the 73 youth whose data are reported here, compared to the larger group (*X*^2^ = 4.495, *p* = 0.034).

#### Consent

All project procedures were reviewed and approved by the Wayne State University Institutional Review Board, specifically by those committees that dealt with research involving vulnerable participants, in this case minors and prisoners (the latter also required U.S. Department of Health and Human Services approval, which was obtained prior to study commencement). Permission for every youth to participate in the Teacher's Pet Program was obtained from the parent or legal guardian of each youth, or from an advocate such as a facility staff member if parental or legal guardian consent could not be obtained, due to absence of a legal guardian. Assent was also obtained from each adolescent who participated. Youth were free to participate or not, and were allowed to stop participating in the program before their portion of the study was completed, although none chose to do so. Compensation for full completion of the Teacher's Pet Program consisted of a $50 Target gift card that was given to each participant after completing the program, when their period of incarceration ended.

### Study Conditions

The Teacher's Pet Program was completed in cohorts, with each cohort participating in training and dog walking that lasted 10 weeks. There were about 10 participants per cohort, with both experimental and control conditions being carried out for each cohort. Youth in each cohort were randomly assigned to either the animal assisted therapy group (Teacher's Pet—learning to train dogs), or the control group (walking the dogs only, for 10 weeks and the same amount of time with dogs as the experimental participants had). Participants in both conditions interacted with dogs for an equal period of time each week, about 2 h total. They also had classroom-based didactic sessions each week that focused on information about dog care, dog behavior, and humane treatment, with youth assigned to both conditions together in the same class periods. Journals were available and journal writing took place during those classroom sessions^1^.

Sessions in which the participants interacted with the dogs occurred for 2 h each week in either an indoor gymnasium, or weather permitting, in an enclosed outdoor courtyard within the facility. Training of dogs occurred in 1 h increments, twice per week, whereas dog walkers either walked assigned dogs for 1 h twice per week or for half an hour 4 times per week. Participants in the experimental group were assigned a dog to train; the main goal for these participants was to train their assigned dog for one half of the program (5 weeks) and were then given another dog to train for the remaining 5 weeks of the program. Dog walkers were assigned different dogs for each session of dog walking, although some youth traded dogs for walking if they chose to do so. These participants were instructed not to train the dogs they walked. Experienced dog trainers or shelter staff were present with the youth and animals through every session of dog-youth interaction, to ensure both fidelity to the assigned activities and safety for the youth and the dogs. Coders were also present to observe participant behavior during training sessions.

### Dogs in the Study

The dogs who participated in this project were brought in daily from nearby Southeast Michigan animal shelters. Facilitators or volunteers for the Teacher's Pet Study worked at the animal shelters and transported the dogs to and from the shelters for each session. No dogs were hurt in the course of their participation in this study. The procedures for the dogs selected for this project were approved by the Institutional Animal Care and Use Committee of Wayne State University. All shelter dogs had a health examination as well as a temperament assessment. Potential dogs for this project also underwent another screening by Teacher's Pet staff to test for major behavioral issues, to ensure the safety of dogs and participants. This screening was essentially similar to that given dogs made available for adoption. Any dog that displayed aggression toward humans or other dogs was immediately ineligible for this study. The dogs included in this study were made up of a variety of breeds, commonly pit bull mixes, and were at least 1 year of age or older. Dogs that displayed minor behavior problems, such as jumping, pulling, and having socialization difficulties, made good candidates for the programs' enrichment and training.

The history of the program was such that dogs trained by the youth had a record of high success in being adopted once they had been trained by Teacher's Pet participants. Although dog adoption was not a specific outcome planned for this project, estimates are that adoption rates for the participating dogs were close to 90%. National estimates of adoption rates for shelter dogs are about 60% (27).

#### Didactics

All participants also engaged in a 1 hour classroom (didactic) session twice weekly, held after training or walking activities. Youth in these classroom activities were not separated by treatment condition, but were grouped together for all class sessions. In the classroom portion of the program, participants learned about dog training techniques, animal shelters, puppy mills, dog behavior, facts about certain dog breeds, etc. Teacher's Pet staff and the staff from the juvenile detention center facilitated the classroom sessions. During the last part of each classroom meeting, the youth were instructed to write in personal journals that were given to them specifically for this program. Participants had either specific writing assignments about varying topics (many relating to the participants' assigned dogs or material in the classroom), or the youth could write freely for this part of the classroom sessions. The youth were also allowed to use the journals to take notes on information that was presented during more didactic classroom portions, if they wished. Although assignments were made or suggested in some didactic sessions, journal content was not graded or evaluated for conformity to any specific instructions given during classroom sessions. The journal content became the primary focus of this study.

### Instruments

Instruments used for the original Teacher's Pet Study consisted mainly of ratings of youth by the facility staff, and self-report measures given to participants pre- and post-intervention. Staff of the detention facilities also reviewed each participant's information and medical chart. Some information for this project was gathered from that review, including participant age, gender, ethnicity, psychiatric and medical history, and history in foster care.

### Analysis of Participant Journal Content

Although all youth in the program (regardless of whether they were in the Teacher's Pet group or the control, dog walking group) had a journal to use during the didactic portion of the study, as noted, not all journals were made available for the project reported here as noted above.

Four raters were assigned to evaluate the content of the journals. Although each had some knowledge of the overall purpose of the project, the ratings that they did were blind to the condition to which journal writers were assigned. Raters did not serve as coders of the live interactions of youth with the dogs. Other than three of the journals that explicitly mentioned which group the writer was in, condition was not obvious from reading the journals. Each rater coded two-thirds of the journals, with each journal being rated by two additional and different raters among the group, through a random process.

### Development of the Coding System

Through a series of analytic steps applied to the journals collected, we devised a coding system for the content of the journals. Originally, it was hoped that writing rated in the journals would represent 6 kinds of categories, including perspective-taking/empathy with others; humane attitudes, attachment to a dog or dogs, self-efficacy/self-perceived competence, emotion regulation of self, and overall reactions to the program. However, when the coders began an initial reading of the journals to orient themselves prior to coding, only some of those categories appeared easily observable, leaving much writing uncategorized.

Therefore, a coding system was developed inductively, beginning with the content of the youths' writing. First, journals were reviewed and topics and content that seemed important or which were mentioned by several youth were noted. These included topics presented freely in participant writing, things clearly appearing to respond to classroom presentations where journaling followed those presentations, and some interesting extraneous material such as drawings. These topics and characteristics were discussed extensively by the group of three coders, until we agreed initially on 50 different types of content or writing characteristics that were observed in the journals. A manual was developed in order to guide the researchers through the process of analyzing and coding individual journals for the 50 types of data that were observed across the entire set of available journals. This manual contained direct and indirect examples of each of the individual coding categories taken from excerpts of the journals themselves in order to help researchers with coding properly. Next, every journal was coded for each of these 50 types of content, on a numerical scale pertaining to how many times a certain category was mentioned or described in writing or observed within each individual journal.

### Reliability of Initial Coding

For reliability purposes, every journal was rated by three raters, with the particular combination of raters determined by a random order designed to produce the same number of raters for each journal, and each rater rating the same number of journals as every other rater. In addition, every journal was rated by a total of three raters, with the configuration of which three persons rated any individual journal across the entire set of journals determined at random. The data obtained from multiple ratings of the same set of journals, were used to calculate pair-wise reliability as well as reliability across three raters. As a result of these ratings, several categories were removed from the rating system, because they were unreliable as coded, or had rarely been observed across the set of journals.

### Obtained Reliability of the Remaining Categories

The obtained average internal consistency of ratings of the remaining categories (which were obtained through Intra-class correlation reliability coefficients (ICC) as recommended by Shrout and Fleiss (28) are found in Table 2. These categories were chosen based on the high range of agreement across three raters as well as the relevance of these categories to the study. Categories below ICC of 0.625 were dropped from further consideration. Individual code categories were then placed into groupings that appeared to be related, based on the content of each code, as described above. Thus, six larger categories were identified and labeled to reflect the items they contained: *future orientation, cognitive growth* and *self-awareness*. These, taken together, appeared to be primarily cognitive codes. Other responses, as coded, involved *attachment, attitude toward the program* and *positivity of emotion*. These together seemed to reflect primarily emotion-related content.

**Table 2:** Sets of coding for participant journal contents.

**Type of code**	ICC^*^
**COGNITIVE CODES**	
**Future orientation**	
FUT Hope for assigned dog	0.95
FUT Participant hope for him- or herself	0.86
**Cognitive growth**	
COG Letter to adopter	0.98
COG Trainer notes	0.96
COG Mentioning dog behavior	0.91
COG Youth says dog has changed youth's attitude	0.63
**Self-awareness**	
SELF Having a relationship with staff	0.98
SELF Showing insight into own behavior	0.93
SELF Comparing self to dog	0.90
SELF What participant learned in program	0.88
**EMOTIONAL CODES**	
**Attachment**	
ATT Attachment	0.99
ATT Physical contact with dog	0.92
ATT Feelings and thoughts of dog	0.84
ATT Wanting to protect dog or other dogs	0.77
ATT Empathy toward dogs	0.73
**Attitude toward the program**	
APROG What participant learned in program	0.88
APROG Liking program	0.82
APROG Training challenge goal assigned	0.73
**Positivity of Emotion**	
LIKE Negative feelings	0.99
LIKE Happy walking or working dog	0.98
LIKE Positive feeling	0.98
LIKE Dog liking participant	0.89
LIKE Critiquing assigned dog	0.81

* *ICC, Intraclass correlation coefficients, using the average of random raters (28)*.

For the emotion-related codes, a group of codes called *Attachment* consisted of participants' writing about their interactions with their assigned dog, such as physical contact, empathy toward their assigned dogs or dogs in general (such as a negative reaction toward a movie on animal shelters), wanting to help dogs, or feeling sorry for their assigned dogs or other shelter dogs, writing about patience, physical contact with their dog (e.g., hugging), talking about having affection for their dog, as well as writing about the feelings or thoughts of the assigned dog. The codes categorized as *Attitude Toward the Program* included writing about the participant's general outlook of the program and what they got out of the program, writing about liking the program, training challenges or goals for their assigned dog, and writing about what they had learned in the program. *Positivity of Emotion*, the label given to another set of codes, included participant writing about their dog liking them, any mention of their own positive feelings (e.g., feeling good or happy) whether related to the program or not, mention of negative feelings whether related to the program or not, a critique of their assigned dog, and writing about being happy about working and/or being with their assigned dog.

For the cognitive-related codes, a group of codes labeled *Future Orientation* included writing about being hopeful of the future for themselves (e.g., writing about careers, what they plan on doing after the program, writing about the future in general, etc.) and being hopeful about the future of their assigned dog(s), e.g., hoping that the dog or dogs get adopted, etc. A set of codes called *Self-Awareness* included participants' writing about their relationship with the staff at the facility, showing insight into their own behavior (e.g., self-reflection), comparing themselves to their assigned dog and vice-versa, writing about what they had learned in the program (also included in the *Attitude Toward the Program* group); and mentioning positive and negative feelings whether the emotions were related to the program or not. Finally, the set of codes called *Cognitive Growth* included such things as a letter to the adopter of a dog, a flier or a story about an assigned dog (a writing prompt given during the classroom portion in the study), the participant writing about how a dog changed the participant's attitude, writing about the observed behaviors of an assigned dog, and presence of notes left by the staff of the program (staff read participants' journals and left notes and/or follow-up questions for some but not all of the youth).

In summary, the codes were organized into larger, labeled sets of codes as described above, with the overall total of the individual ratings for each set serving as data for further analysis. Table 2 contains interrater reliability coefficients for the codes making up each set.

### Educational Level of Writing

It was reasonable that the sophistication or general education level of the journal writing could also have an effect on what youth wrote (29, 30), given that incarcerated youth often show academic deficits. It was also the case that no analysis of educational level was made in the larger project from which this study data had been obtained, nor did youth records indicate their educational level. Thus, it was decided to estimate youths' writing/education level, to make it available for analyses of experimental vs. control group differences in journal content. Participant journals were therefore rated based on overall written sophistication observed in each journal. Two expert raters with extensive background knowledge and teaching experience working with young writers and students made holistic ratings of each journal in determining educational sophistication based on journal writing content and detail. These raters had not met nor observed any of the participants during the intervention and were unaware of the group to which the youth writers had been assigned. Both raters rated every journal. Ratings were based on the general written sophistication of content in the journal entries, using a scale of 1–3, with “1” being lowest, “2” medium, and “3” highest in written sophistication. The inter-rater agreement for the two raters was 0.75 (Cohen's Kappa) across the full set of journals. The ratings of the two raters for each participant were summed, producing scores ranging from 2 to 6, with lower scores representing less writing skill or educational sophistication than higher scores. These scores were used in the data analysis to take into account possible effects, if any, of writing/educational sophistication on the content of the journals' content ratings.

After writing level scores were obtained, relations to writing content sets were analyzed. Education sophistication level had significant positive correlations with every set of codes, with higher writing levels associated with higher scores in the content categories. These relationships of general writing/educational level to content categories included Cognitive Growth; *r*(71) = 0.46, *p* < 0.01; Future Orientation; *r*(71) = 0.50, *p* < 0.01; Self-Awareness; *r*(71) = 0.49, *p* < 0.01; Attitude Toward the Program; *r*(71) = 0.38, *p* < 0.01; Positivity of Emotion *r*(71) = 0.29, *p* < 0.05; and Attachment *r*(71) = 0.40, *p* < 0.01. Thus, it was decided that educational sophistication would be used as a covariate in subsequent analyses of differences between experimental and control groups^2^.

### Hypotheses

It was hypothesized that there would be significant between group differences for all 6 areas of journal content, whether Emotional (Attachment, Positivity of Emotion, Attitude Toward the Program) or Cognitive (Future Orientation, Cognitive Growth and Self-Awareness), in that participants in the Teacher's Pet group would have significantly higher average scores compared to participants in the dog walking group.

A MANCOVA (Multivariate Analysis of Covariance) was conducted in order to determine if there were significant differences between the journal content of treatment vs. control groups in terms of Cognitive Growth, Future Orientation, Self-Awareness, Attachment, Positivity of Emotion and Attitude Toward the Program seen in their journal entries, controlling for education sophistication level.

## Results

### Group Differences

Table 3 reports the comparisons and effect sizes for each final code. As expected, there was a statistically significant difference between treatment vs. control groups in means for Cognitive Growth scores, [*F*_(1, 71)_ = 11.32, *p* = 0.05, Wilk's Λ = 0.84, partial η^2^ = 0.16], in that the participants in the Teacher's Pet group had a significantly higher average Cognitive Growth score (*Mean* = 8.05, *SD* = 3.93) compared to the Dog walking group (*Mean* = 4.70, *SD* = 2.52), with an effect size for the intervention of *g* = 0.98, a large effect. Although group differences in Future Orientation scores were not significant, the effect size was moderate, *g* = 0.60, [*F*_(1, 71)_ = 3.65, *p* = 0.06]. Self-Awareness scores, however, did not show group differences at or close to the conventional *p*-level, and did not produce a meaningful effect size [*F*_(1, 71)_ = 0.44, *p* = 0.51, *g* = 0.06]. Thus, in terms of cognitive writing as a function of group membership, changes in Cognitive Growth and Future Orientation scores, favored the Teacher's Pet group, with moderate to large effects.

**Table 3:** Comparisons of experimental vs. control group for journal content codes.

**Coding type**	**Experimental group mean (SD)**	**Control group mean (SD)**	**Effect size**	***p***
**COGNITIVE**				
Future orientation	6.13 (2.91)	4.33 (3.15)	0.60	*p* = 0.06
Cognitive growth	8.05 (3.93)	4.70 (2.52)	0.98	*p* = 0.05
Self-awareness	3.14 (1.27)	3.04 (1.90)	0.06	*p* = 0.51
**EMOTIONAL**				
Attachment	6.79 (5.29)	3.13 (3.10)	0.81	*p* = 0.05
Attitude toward program	3.12 (2.63)	1.08 (0.95)	0.97	*p* = 0.05
Positivity of emotion	4.03 (1.21)	2.97 (2.64)	0.58	*p* = 0.07

*Higher Means signify larger outcomes as seen in the coded measure of journaling content. Effect sizes were calculated using Hedge's g; its computation takes into account both differences and variation between treatment groups. It is noteworthy that strict use of p ≤ 0.05 would eliminate consideration of a medium effect size, that is likely important*.

In terms of emotional aspects of their journal writing as seen in the emotion category scores, there were meaningful effect sizes favoring the Teacher's Pet treatment, in all three sets of coded writing (see Table 3). Scores for Attitude Toward the Program [*F*_(1, 71)_ = 12.67, *p* = 0.05], were significantly higher for participants in the Teacher's Pet group (*Mean* = 3.12, *SD* = 2.63) compared to participants in the dog walking group (*Mean* = 1.08, *SD* = 0.95), effect size of *g* = 0.97, a large effect. There were also significant differences on scores of Attachment for the two groups [*F*_(1, 71)_ = 7.28, *p* = 0.05, Wilk's Λ 0.82, partial η^2^ = 0.18], in that participants in the Teacher's Pet group showed significantly higher scores for Attachment (*Mean* = 6.79, *SD* = 5.29), compared to participants in the dog walking group (*Mean* = 3.13, *SD* = 3.10), with an effect size that was moderately large, *g* = 0.81. The group differences for Positivity of Emotion, [*F*_(1, 71)_ = 3.43, *p* = 0.07], though not conventionally significant, were just outside that level. In addition, the effect size for Positivity of Emotion was *g* = 0.58, a moderate effect. Thus, for ratings of emotional journal writing, then, youths' journal writing in the experimental group showed more positive attitudes about the animal assisted intervention they were doing, stronger attachment to dogs and other living things, and had more positive emotions in general than were observed in writing of the control group. Effects for the set of emotion scores ranged from high to moderate in size.

Overall, the Teacher's Pet intervention demonstrated effects seen through youths' journal writing that ranged from moderate to large in both emotional and cognitive ratings of their writing, with somewhat more differences visible in emotional compared to cognitive ratings of their journal entries.

## Discussion

The results indicate that there were significant differences between the Teacher's Pet (AAT) group and the Dog walking control group in the rated emotional as well as cognitive content of what participants wrote in their journal entries, with youth in the experimental group showing more positive outcomes. This result was somewhat surprising, given that the behavioral outcomes of the youth in the project did not show differences between the experimental and the control group (1). Both groups demonstrated increases in empathy and internalizing problems in analyses of the larger group of youth from which the journals reported here came.

A key question is why the journal writing revealed positive outcomes for treatment that were not seen in behavior ratings made by center personnel nor the ratings that youth gave themselves. Scores based on different journal content pertaining to all categories of codes other than Self-Awareness were noteworthy in showing medium to large effect sizes. Thus, the writing of the youths showed treatment-related positive outcomes in Cognitive Growth, Attitude Toward Being in the Program, Attachment, Future orientation, and Positivity of Emotion. Participating in the Teacher's Pet intervention produced more positive effects, seen in youth journal writing, compared to the dog walking group.

One explanation for their higher average scores is that participants in the Teacher's Pet group were more likely to write adoption flyers, letters to potential adopters of their assigned dog as well as stories about their assigned dog. This writing prompt was given to all the participants during a classroom portion, before the dogs' “graduation day,” at the end of the treatment period. However, because participants in the experimental group worked very closely with just two dogs, this kind of writing may have been more likely for them to do and do better than youth in the dog walking group, who walked several dogs.

The Teacher's Pet Staff also left notes in some of the participants' journals pertaining to any follow-up questions that a participant had, or a youth's journal content, or his or her progress in the program. This was a potential confounding factor for Cognitive Growth, as the staff knew the groups to which the participants were assigned. They may have been more likely to leave notes for participants in the experimental group regarding their progress in the program, which could have also accounted for the higher score for this category. However, no differences in the number of staff comments were noted between the two groups, however, though content of those comments were not evaluated separately.

Participants in the Teacher's Pet group had closer and very different kinds of interactions with their dogs than the dog walking group had, due to training their assigned dogs for the period of the study. Thus, experimental participants were more likely to be aware of and write about the behavior of their particular assigned dogs, compared to dog walkers who may have observed more general dog behavior seen in several animals. In addition, the training itself may have caused the experimental group to track their dog's problems as well as positive behaviors closely, more than would have been the case for the dog walkers. Learning about dog behavior during the educational portion of the study and applying that to their observations while working with their assigned dog(s), and writing about it, could have enhanced their understanding more than would have been the case for the dog walking group. This is consistent with the findings that journaling has helped individuals in therapy gain better understanding of their own behaviors and behaviors of others, and can aid in self-reflection and better overall treatment outcomes (23, 24).

Participants in the Teacher's Pet group also showed significantly higher scores for both Attachment and Attitude Toward Program. The Attachment code included components such as having empathy for the dog, physical contact, affection for their assigned dog), writing about dogs' feelings or thoughts, writing about patience, and wanting to help their own dog or other dogs in general. The higher scores for attachment seen in writing by the Teacher's Pet group could be because those participants spent more time training a specific dog and learning and being more aware of their dog's characteristics and behavior problems. This in turn could have facilitated a strong bond with the dog, possibly aiding their understanding of their dog's feelings or thoughts and promoting feelings of empathy as well as more attachment to their dog. Through this, these participants may also have been more understanding toward these dogs and their situation as shelter animals without homes, as well as writing about patience through the process of training a dog and changing its behaviors. Youth who walked dogs did not have such long-lasting relationships with particular dogs; they typically handled more dogs without being able to stick with just one animal for several sessions, thus their attachment to and knowledge of particular animals was less likely to occur. However, that potential explanation is countered by the finding in the larger project that both groups of youth increased in a formal rating of their empathy, which ordinarily might be expected to be related to content of their writing (1).

In addition, participants in the Teacher's Pet group also showed significantly higher ratings for their Attitude Toward the Program, seen in positive statements such as writing about liking the program, what they learned, and training challenges and goals for their assigned dogs, compared to youth in the dog walking group. Perhaps participants in the Teacher's Pet group were more likely to write and reflect on what they learned in the program through training their assigned dogs. These participants, compared to the control group, were more likely to set goals regarding training or problem behaviors of the dogs. Thus, these participants likely wrote more about what they got out of the program through this process. This is consistent with research findings (31, 32) that writing about what occurs in therapy and reflecting on it can help individuals gain insight and process what is happening in treatment and what benefits them.

There were no meaningful effect sizes Self-Awareness, which typically consisted of writing about having a relationship with the staff at the facility, showing insight into their own behavior, comparing themselves to their assigned dogs and vice-versa, writing about what they learned in the program or mentioning emotions regardless of whether the emotions were related to the program. All participants were asked to write about how their day went, and scoring of positive and negative emotions in the journals was done whether participants wrote about their emotions related to the program or some other topic. This writing prompt may have provided an opportunity for self-reflection for participants in both groups. Moreover, both groups attended the classroom portion of the study and could have reflected on class activities, which did not vary by group.

There is another puzzling aspect to the journal writing. The journal entries were made throughout training, not just at the end of the intervention. Thus, differences in writing as a function of receiving the intervention had an effect that occurred during the intervention, not necessarily as the overall final outcome of the intervention. If the journaling content reveals inner effects of the animal assisted intervention, those effects begin earlier and may build, before behavioral changes are observable.

It may also be worth considering that the original categories that the coders thought would be found in youths' writing were not usable. The coders were not highly familiar with the intervention, and may have also lacked knowledge of adolescent characteristics, or made inaccurate assumptions about the youth in the study. Coder characteristics are not often studied and might have influenced their original ideas about what journal content would include. Future studies should look more closely at rater and coder characteristics to see how they match with what youth actually write about, as reactions to an intervention. Facility staff, who had a lot of contact with youth and thus presumably knew them well, did not see changes, but unfamiliar coders did see signs of change in the writing of the youth, that accorded with their group assignment. Facility staff ratings focused on behavior, whereas the writing more commonly reflected youths' internal thoughts. Changes in thinking and emotion may precede behavioral changes, or be unavailable to persons who have no access to youths' thoughts that could be found in their writing. Perhaps familiarity with youth being rated could alter rater or coder responses; this might particularly be the case among facility staff, where a great deal of disapproval and possible stigma might be attached to expectations of youth. Such negative expectations might not be present with a more neutral assessment, where raters and coders know only a little of the court-ordered placement of the youth.

Despite there being some areas of journal content that were similar for both groups, the results showing treatment effects offer interesting insights into the intervention. The journal content of participants in dog training therapy were rated more positively, on average, for several aspects of that writing. This underscores the idea that youth engaging in an animal-assisted therapy intervention may show how the intervention has increased attachment, empathy, patience, and awareness of what youth have learned, through what they write about. Our findings highlight how journaling can demonstrate progress through treatment, and show attitudes, thought patterns, and emotions that many who work with troubled youth think are valuable outcomes, but which are very hard to observe in youth behavior. If so, this suggests that knowing the content of expressive writing of youth as they work with animals, may reveal the depths of how human-animal interaction influences the adolescents in a positive direction, before behavior change is evident (18).

### Limitations

There were several limitations to this study. The number of participant with journals rated was smaller than the number of youth in the larger project. Although youth whose journals were available for rating appeared to be similar to the larger group, the missing journals cannot be ruled out as a cause of the differences in outcomes for this study compared to the larger project. It is possible, though not likely, that the writing of the youth whose journals were not available for analysis was sufficiently different from the journals analyzed for this report, that the group differences would not be present had all journals been rated.

It is not clear how these results are related to the outcomes of the larger study. It would be ideal had all the journals been analyzed for content, allowing direct comparison of outcomes for the full number of participants who received the intervention or the control. In addition, there was no follow-up of these participants. It is not known whether the positive outcomes seen in the writing of the experimental group do in fact predict better longer term outcomes, such as lower rates of recidivism. The youth in this study, while being generally representative of the facilities from which they were drawn, are not known as to what degree they represent at-risk incarcerated youth nationwide. The AAT, Teacher's Pet, in this project, is a long-running, well-developed intervention that has highly trained staff. Other animal assisted interventions may not have the same degree of training and experience, which could be keys to success of treatments in general for mental health and behavior problems.

Finally, the uneven randomization due to facility constraints may have impacted the results, even though the group sizes for the 73 youth participant in this examination of journal writing, were more balanced than was the case for the 138 youth in the original project. This limitation is a function of conducting research with community partners, for which programmatic, staffing, and scheduling resources are natural priorities. Similar imbalances favoring more youth assigned to treatment are common in studies of conduct disordered youth (26). Researchers must continue to balance internal and external validity needs with the reality of conducting real-world investigations that can be affected by many competing priorities.

### Implications and Recommendations

Given the simplicity of including journaling as a way to assess changes due to treatment, and the relatively low cost of animal assisted interventions, programs such as Teacher's Pet show promise for expanding the range of interventions for incarcerated youth. It is also worth assessing for use with persons, including youth, who for other reasons cannot receive the typical model of therapy that rests on one person with one therapist at a time, over an indefinite period. The efficiency of group interventions along with youth attraction to dogs, makes such interventions an attractive option for incarcerated youth, as well as community based treatment programs.

Recommendations for future research include studying the journal content of additional at-risk adolescents engaged in a similar intervention and analyzing common themes in the journals along with self-reported scores for behavioral problems pre and post-assessment, in order to evaluate behavioral progress. It could be beneficial to interview participants pre and post-assessment in order to assess how the content of their writing relate to other youths' perceptions of their responses to intervention.

As it was primarily undesirable behavior that caused the youth in this project to be incarcerated, more study is needed to see whether animal-assisted interventions are effective in changing such youths' behavior after they are released. Using journaling as an adjunct to assessing other outcomes of treatment could show changes in motivations and attitudes that lead to successful post-incarceration adjustment. Long-term follow-up is therefore an essential need for future studies. Only by seeing whether the positive effects such as those found here in journal writing predict a lack of recidivism will it be clear that such interventions, assessed through journal writing, are accurately predictive of long term positive outcomes.

The findings of this study highlight the potential usefulness of journaling as a method for the assessment of treatment. This could be particularly valuable as a way to evaluate an active, very participatory intervention such as the animal-assisted therapy featured in this study, in which the participants trained dogs or worked closely with them. By analyzing the content of the journals of these participants, it appears that this animal-assisted therapy facilitated empathy, attachment, behavioral insight, and patience in the youth, outcomes that were shown only by examining the writing of the youth in treatment. This demonstrates that animal-assisted therapy can facilitate some changes in cognitive as well as emotional attitudes in youth that are not readily observable in their overt behavior. Those changes in turn can extend into improved interpersonal relationships and empathy toward others among the youth, after the intervention is over (33–35).

Journaling can be a cost-effective and useful way to assess the effects of treatments given to vulnerable adolescents, facilitating knowledge of treatment outcomes in a population that is still developing cognitively, who may not show therapeutic changes visibly, although changes are resulting from the treatment. Similar interventions including animals as a key feature of the treatment along with journaling as an assessment of responses to therapy, are promising for the possibility that they can ultimately show that treatments reduce recidivism and improve the quality of life for at-risk youth.

## Author Contributions

RC was one of the primary leaders of the original study, co-principal investigator of the NIH grant that funded the project. AC was the principal investigator of the NIH grant for the original study. AJ was one of the co-principal investigators of the original, NIH funded project. She is the leader of the Teacher's Pet AAT program, which was central to the treatment condition in this project. TS was a research assistant to this project. She was central to the development and execution of the journal coding system reported in this project. NS was a graduate research assistant to this project, he analyzed data and reported on the larger project from which this study was developed. DA was a graduate research assistant to this project. She was involved in the planning of the project, and was a leader in the collection of data and interaction with shelter staff and coders for the project.

### Conflict of Interest Statement

The authors declare that the research was conducted in the absence of any commercial or financial relationships that could be construed as a potential conflict of interest.

## Footnotes

^1^Additional details concerning the content of the Teacher's Pet intervention can be obtained from the authors upon request, or by going to the web site associated with the intervention (www.teacherspetmi.org). See Author Notes for information about contacting the authors.

^2^Data from the study are available on request from the authors.
